# Beware of Bogus Dentists

**Published:** 1886-06

**Authors:** 


### Beware of Bogus Dentists.

In the *American Agriculturist* C. P. Dewey says that the operation of pulling teeth is not ordinarily regarded as an "entertainment," either by the dentist or his victim. Yet it was made so by a "professor" in Reading, Pa., who was arrested there a few days ago. He called himself a painless tooth extractor, and had a brass band and a variety singer, who acted as a popular allurement and hundreds of people were enticed into the operating establishment and there induced to part with their teeth, and then with their money, for worthless medicine and more worthless advice. There is a class of traveling and advertising dentists who go about the country and practice their art upon credulous people, in ignorance of its principles, whose sufferings in the end are only heightened by the mistaken means they have taken for relief. The art of dentistry has made a great advance within a few years, and what was once deemed impossible is now easily accomplished. It is probable that the time is not distant when to draw a tooth because it aches will be regarded as absurd as to lop off a finger because it is painful. Teeth are treated and "doctored" with as much system and success as any other part of the organization, and are preserved to a good old age as assuredly as an eye or an ear. But they must be examined and inspected by a competent dentist every year or two, to discover whether there are signs of decay, and to remove the deposits of tartar which may threaten them. Such an examination is simple and cheap, and saves much pain and cost in the end. Of course no one should neglect the daily habit of using a tooth-brush, by which means the clinging particles of food and the minor accretions that disfigure the teeth are removed. Nothing adds more to the good looks of a man or woman than well-regulated teeth, and nothing is more unbecoming than a mouth with bad, or broken, or discolored teeth, the relics and ruins of what might have been ornamental and useful. It is a very great comfort to possess good teeth to the end of life, and the comfort is worth paying for. If properly taken in hand early in life, and when the first indications of decay appear the teeth may be usually made secure for all time.

